# Fathers’ involvement in child feeding and associated factors among fathers of children aged 6–24 months in Chena District, Southwest Ethiopia: a community-based cross-sectional study

**DOI:** 10.1038/s41598-026-40365-1

**Published:** 2026-02-15

**Authors:** Daniel Mulat Eshetu, Mikias Getahun Molla, Zebene Ambaw

**Affiliations:** 1 Department of Public Health, College of Medicine and Health Sciences, Injibara University, Injibara, Ethiopia; 2 Department of Public Health, College of Medicine and Health Sciences, Mizan Tepi University, Mizan Aman, Ethiopia

**Keywords:** Fathers’ involvement, Child feeding, Children 6–24 months, Chena district, Diseases, Health care, Medical research, Risk factors

## Abstract

Fathers’ involvement in child feeding positively influences feeding practices and child nutrition. Inadequate feeding practices are a major contributor to preventable childhood illnesses and mortality. Although fathers’ participation is important, evidence on the level and factors associated with their involvement in the study area is limited. This study aimed to assess the magnitude of fathers’ involvement in child feeding and associated factors among fathers of children aged 6–24 months in Chena district, Kaffa zone, Southwest Ethiopia, 2025. A community-based cross-sectional study was conducted from February 1 to June 20, 2025, among 622 fathers of children aged 6–24 months, selected using systematic random sampling. Data were collected via structured, pretested questionnaires, coded, entered into EpiData v4.6, and analyzed using SPSS v27. Variables with *p* < 0.25 in bivariable analysis were included in multivariable logistic regression. Adjusted odds ratios (AOR) with 95% confidence intervals (CI) were calculated, and *p* < 0.05 was considered statistically significant. Of the 622 fathers, 259 (41.6%) were involved in child feeding. Factors positively associated with involvement included college education or above (AOR = 4.41, 95% CI: 1.67–11.66), good knowledge of child feeding (AOR = 2.84, 95% CI: 1.78–4.51), positive attitude (AOR = 2.10, 95% CI: 1.34–3.28), and good cultural beliefs regarding child feeding (AOR = 2.22, 95% CI: 1.40–3.51). Fathers whose workplaces were far from home and who were mostly absent at night were less likely to be involved (AOR = 0.44, 95% CI: 0.22–0.90). Fathers’ involvement in child feeding in Chena district is low. Educational status, workplace proximity, knowledge, attitude, and cultural beliefs significantly influence fathers’ engagement. Interventions promoting father participation should address these factors to improve child feeding practices.

**Supplementary Information:**

The online version contains supplementary material available at 10.1038/s41598-026-40365-1.

## Introduction

Fathers’ involvement in child feeding encompasses observable actions that positively influence feeding practices, including financial, physical, and resource support; shared responsibility; and psychological and social support to mothers^1^. Evidence from Ethiopia shows that paternal engagement increases household dietary diversity among children aged 6–23 months by 13.7%, highlighting the importance of promoting fathers’ direct participation in child feeding^2^. Father involvement is also associated with improved infant and young child feeding (IYCF) practices and positive emotional, developmental, and health outcomes in children^3,4^.

Proper child feeding during the first two years is critical for optimal growth, health, and development, yet this period is often marked by micronutrient deficiencies and frequent childhood illnesses^5^. IYCF practices, including timely introduction of complementary foods at six months, appropriate feeding frequency, and dietary quality, directly affect child nutritional status and survival^6–8^. While mothers are primarily responsible for IYCF, interventions are less effective without engaging fathers^9–12^. Evidence from Vietnam, Peru, sub-Saharan Africa, and South Africa indicates that children whose fathers are not involved in feeding are at higher risk of malnutrition^13^.

Despite the recognized benefits, most research and interventions have focused on mothers, whereas fathers’ roles, particularly in developing countries, remain largely neglected, despite their influence on household decision-making^12,14–16^. In Ethiopia, fathers traditionally manage resources while mothers handle childcare and domestic tasks; however, optimal child development depends on care from both parents^17,18^. Recommended feeding practices for children aged 6–23 months include continued breastfeeding up to two years and timely complementary feeding with diverse and frequent meals, which are associated with reduced undernutrition, lower mortality, and improved developmental outcomes^19,20^.

Globally, malnutrition contributes indirectly to over 33% of infant deaths, with 5.4 million under-five deaths in 2021, roughly half occurring in sub-Saharan Africa^9,21^. In Ethiopia, 38% of children are stunted, 10% wasted, and 24% underweight^22^, reflecting both acute and chronic undernutrition, which accounts for more than 50% of child deaths in developing countries^23^.

Fathers often contribute financially but less frequently in direct caregiving or emotional support^24^. Poor paternal involvement affects mothers’ ability to follow recommended feeding practices and increases children’s risk of growth faltering, poor cognitive development, and social problems^25–27^. Factors influencing low paternal involvement include inadequate knowledge, cultural beliefs, attitudes, low education, age, employment, limited social support, and inequitable household decision-making^24,28^.

In Southwest Ethiopia, including the Chena district, data on fathers’ involvement in child feeding are limited, and interventions promoting paternal engagement remain sparse. Chena district was purposively selected because it is representative of rural communities in Southwest Ethiopia, has a high prevalence of child undernutrition, and has limited data on fathers’ involvement in child feeding. Conducting the study in this district allowed for feasible community-based data collection while addressing an under-researched area. Therefore, this study aimed to assess fathers’ involvement in child feeding and associated factors among fathers of children aged 6–24 months in the Chena district, Kaffa zone. Understanding fathers’ involvement can enhance initiation and continuation of optimal feeding practices and guide health-care providers and local health offices in planning interventions to reduce suboptimal child feeding and improve child health outcomes.

## Methods and materials

### Study area and period

The study was conducted in Chena district, Kaffa Zone, Southwest Ethiopia, from February 1 to June 20, 2025. The Chena district, located 510 km southwest of Addis Ababa along the main road to Mizan, comprises 24 kebeles and is primarily rural. The local economy is based on agriculture and animal breeding, with fertile land suitable for crops such as coffee, teff, wheat, and banana. According to the 2025 District Health Sector Transformation Plan (HSTP), the district has a population of 99,951, including 3,508 children aged 6–23 months, representing 20% of households. Health facilities include three health centers, one primary hospital, twenty-four health posts, fifteen private clinics, four medium clinics, and two drugstores (Chena District HSTP, 2025).

### Study design

A community-based cross-sectional study was employed.

### Population

Source population: All fathers who had children aged 6 to 24 months residing in Chena District 

Study population: Fathers with children aged 6–24 months residing in the selected kebeles during the data collection period who fulfilled the inclusion criteria.

### Sample size determination

The sample size for the first objective (magnitude of fathers’ involvement) was calculated using a single population proportion formula, assuming a 95% confidence interval, 5% margin of error, design effect of 1.5, and a prevalence of fathers’ involvement of 43.1% from Antsokia Gemza District^1^. The minimum sample size was 566; after adding 10% for non-response, the final sample size was 623.

For the second objective (factors associated with fathers’ involvement), sample sizes were calculated using EPI-Info 7.2.6 based on expected proportions of exposed and unexposed groups, 95% CI, 80% power, and a 1:1 ratio (Table 1).

**Table 1:** Sample size determination for factors associated with fathers’ involvement in child feeding among fathers with children aged 6–24 months in Chena district, Southwest Ethiopia.

Variable	% Non-exposed	% Exposed	AOR	Total Sample Size	Reference
Fathers’ health facility visits	58.2%	89%	5.84	84	^29^
Fathers’ good knowledge	14.3%	39.1%	3.84	125	^1^
Fathers’ positive attitude	12.6%	55.2%	8.56	51	^1^

### Eligibility criteria

Fathers of children aged 6–24 months who lived in the study area with the child’s mother and were actively involved in the child’s life, regardless of biological or adoptive status, were eligible for inclusion. Fathers with critical health problems or those who did not live with the child’s mother were excluded from the study.

### Sampling procedure

A multi-stage sampling technique was used to select study participants. First, all 24 kebeles in Chena district were stratified, and 8 kebeles (representing 30% of the total kebeles, as recommended by WHO) were randomly selected using a lottery method. The calculated sample size was proportionally allocated to each selected kebele based on the number of fathers with children aged 6–24 months, as identified from family folder lists and growth monitoring registers at health posts. The total number of fathers with children from 6 to 24 months placed in Table 2.

Systematic random sampling was then applied within each kebele to select individual participants. The sample interval was determined using $\:K=N/n=1365/622\approx\:2$, where N is the total number of fathers in the selected kebeles and n is the total sample size. The first father in each kebele was selected randomly by lottery from the first two eligible fathers, and subsequent participants were selected at every second interval (2nd, 4th, 6th, etc.) until the allocated sample for each kebele was fulfilled. Fathers’ names and addresses were obtained in collaboration with kebele health extension workers and health development army leaders, and interviews were conducted in their homes.

**Table 2:** Number of fathers with children aged 6–24 months in selected kebeles.

Kebele	Donga	Kulush	Koda	Shacha	Sheka	Wota	Aja Bamba	Gaygoy
Number of fathers that had children aged 6–24 months	168	56	244	223	167	138	188	181

The proportional allocation of the 623 participants to each kebele was calculated using the formula $\:{n}_{i}=(n/N)\times\:{N}_{i}$, where $\:{n}_{i}$is the sample size for each kebele and $\:{N}_{i}$is the number of fathers in that kebele. Accordingly, the allocated sample sizes were: Donga = 77, Kulush = 26, Koda = 111, Shacha = 101, Sheka = 76, Wota = 63, Aja Bamba = 86, and Gaygoy = 83.

### Operational definitions

Fathers’ involvement: Fathers’ involvement was measured using a composite score derived from multiple Likert scale items. Participants who scored above 50% of the overall mean score were categorized as having good involvement, while those with scores of 50% or below were classified as having poor involvement^1^.

Knowledge: Good knowledge = score above mean; Poor knowledge = score at or below mean^1^.

Attitude: Positive attitude = score above mean; Negative attitude = score at or below mean^1^.

Cultural belief: Good cultural belief = score above mean; Bad cultural belief = score at or below mean^1^.

### Measurement

Fathers’ involvement in child feeding: Assessed using 13 items across five domains (shared decision-making, physical support, psychosocial support, financial/resource support, and workload sharing) on a 4-point Likert scale (1 = never to 4 = always). Total score above the mean = good involvement; at or below the mean = poor involvement^1^.

Knowledge: Measured using 9 multiple-choice items on complementary feeding, breastfeeding, dietary diversity, feeding frequency, fathers’ roles, and consequences of poor nutrition. Correct = 1, incorrect/don’t know = 0. Total score ≥ 5 = good knowledge; <5 = poor knowledge^1^.

Attitude: Measured using 8 Likert-scale items (1 = strongly disagree to 5 = strongly agree) on satisfaction with breastfeeding, household support, meal preparation, childcare, and nutrition involvement. Total score above mean = positive attitude; at or below mean = negative attitude^1^.

Cultural belief: were assessed using five yes/no items that captured discouragement or support from the community, family, and peers. Responses were coded as 1 for favorable beliefs and 0 for discouraging beliefs. Fathers with a total score of 3 or higher were classified as having good cultural beliefs, while those with a score below 3 were classified as having bad cultural beliefs^1^.

### Variables

Dependent variable: Fathers’ involvement in child feeding.

Independent variables: Socio-demographic factors (age, residence, marital status, family size, father and mother education and occupation, parenthood, income, sex of youngest child, sources of information, workplace, and paternity), knowledge about child feeding, attitude toward involvement, and cultural beliefs regarding involvement.

### Data collection tools and procedures

A structured questionnaire was developed after reviewing relevant literature. It consisted of items on sociodemographic characteristics as well as questions assessing fathers’ knowledge, attitude, cultural beliefs, and involvement in child feeding practices. The questionnaire was initially prepared in English, then translated into Amharic by language experts. A separate expert translated it back into English to ensure consistency. Data were collected through face-to-face interviews during house-to-house visits. Fathers of children aged 6 to 24 months were interviewed, and those who were not available after three consecutive visits were excluded and replaced by the next eligible household to achieve the required sample size.

### Data quality control

Data were collected through face-to-face interviews using a structured questionnaire developed from previous literature. Three diploma nurses and one supervisor with a Bachelor of Science degree were recruited to conduct and oversee the data collection. A two-day training was provided covering the study objectives, the importance of the research, informed consent procedures, confidentiality, and interview techniques. A pretest was conducted on 5% of the total sample size (31 participants) in a different kebele (Dosha Tuga) prior to the actual data collection to assess the clarity, comprehension, and appropriateness of the questionnaire. The tool used to measure fathers’ involvement had been previously validated in this setting. Necessary modifications were made based on the pretest findings to ensure data quality.

### Data processing and analysis

The collected data were checked for completeness and consistency before entry. The data were coded, entered, and cleaned using EpiData version 4.6, and then exported to SPSS version 27 for further analysis. Descriptive statistics including frequencies, proportions, and summary measures were computed to describe the study population in relation to key variables and were presented using tables.

Bivariable binary logistic regression analysis was conducted to identify candidate variables for multivariable analysis using a p value threshold of < 0.25. Variables that fulfilled this criterion were entered into a multivariable binary logistic regression model to identify factors independently associated with fathers’ involvement in child feeding. Adjusted odds ratios (AORs) with their corresponding 95% confidence intervals were calculated to assess the strength of associations. Statistical significance was declared at a p value < 0.05. Model fitness test was checked by Hosmer-Lemshow (HL) goodness of fit test, where a p value > 0.05 indicating a fitted model. Multi-collinearity was checked by Variance inflation factor (VIF), where a VIF > 10 indicating the presence of multi-collinearity.

## Results

### Socio-demographic characteristics

A total of 622 fathers with children aged 6–24 months participated in the study, yielding a response rate of 99.84%. This was achieved through multiple contact attempts; a participant was considered non-responsive if absent after three consecutive visits. The majority of fathers, 245 (39.4%), were aged 31–40 years. Most participants, 544 (87.5%), resided in rural areas, and 576 (92.6%) reported monogamous marital status.

Regarding exposure to information about fathers’ involvement in child feeding, 565 (91%) of fathers reported having heard about it. Of these, 190 (30.5%) received information from mass media, while 222 (35.7%) heard about it from health facilities (Table 3).

**Table 3:** Socio-demographic characteristics of fathers’ involvement in child feeding among fathers with children aged 6–24 months in Chena district, Southwest Ethiopia.

Variable	Category	Frequency (*n*)	Percent (%)
Age of fathers (years)	18–30	160	25.7
	31–40	245	39.4
	41–50	154	24.8
	> 50	63	10.1
Residence	Urban	78	12.5
	Rural	544	87.5
Marital status	Monogamy	576	92.6
	Polygamy	46	7.4
Family size	Three	118	19.0
	Four	198	31.8
	Five and above	306	49.2
Father educational level	No formal education	127	20.4
	Primary education	233	37.5
	Secondary education	144	23.2
	College and above	118	19.0
Father occupational status	Government employee	166	26.7
	Merchant	115	18.5
	Farmer	311	50.0
	Daily laborer	30	4.8
Mother educational level	No formal education	223	35.8
	Primary education	137	22.0
	Secondary education	108	17.3
	College and above	154	24.7
Parenthood	First-time father	355	57.1
	Experienced father	267	42.9
Paternity	Biological father	560	90.0
	Non-biological father	62	10.0
Source of information	Mass media (TV or radio)	190	30.5
	Social media	23	3.7
	Health facility	222	35.7
	Neighbor/community	131	21.1
	Have not heard information	56	9.0
Monthly average income (ETB)	< 1500	235	37.8
	1501–2500	121	19.5
	2501–3500	117	18.8
	> 3500	149	24.0
Sex of youngest child	Male	327	52.6
	Female	295	47.4
Work place of fathers	Near residential area, night at home	466	74.9
	Far from residential area, sometimes not home at night	71	11.4
	Far from residential area, mostly not home at night	85	13.7

### Fathers’ knowledge, attitude, cultural belief, and involvement in child feeding

Among fathers of children aged 6–24 months in Chena District, 60.0% had good knowledge of child feeding practices. Positive attitude toward child feeding was observed in 41.3% of fathers. Favorable cultural beliefs supporting involvement were reported by 53.5% of fathers. Overall, 41.6% of fathers were classified as having good involvement in child feeding (95% CI: 37.7%–45.5%) (Table 4). Detailed item-level responses are presented in Supplementary Tables S1–S4.

**Table 4:** Summary of fathers’ knowledge, attitude, cultural belief, and involvement in child feeding among fathers of children aged 6–24 months in Chena District, Southwest Ethiopia.

Domain	Overall category	Frequency (*n*)	Percentage (%)
Knowledge	Good	373	60.0
	Poor	249	40.0
Attitude	Positive	257	41.3
	Negative	365	58.7
Cultural belief	Good	333	53.5
	Poor	289	46.5
Fathers’ involvement	Good	259	41.6
	Poor	363	58.4

Knowledge, attitude, cultural belief, and involvement were assessed using structured questionnaires.

Item-level responses are provided in Supplementary Tables S1–S4.

### Factors affecting fathers’ involvement in child feeding

Bivariable analysis identified age, marital status, educational level, occupational status, paternity, source of information, monthly income, sex of the youngest child, workplace, knowledge, attitude, and cultural belief as candidate variables (*p* < 0.25) for multivariable analysis. In multivariable logistic regression, educational level, workplace, knowledge, attitude, and cultural belief were significantly associated with fathers’ involvement. Fathers with a college-level education or above had 4.41 times higher odds of involvement compared with those with no formal education (AOR = 4.41, 95% CI: 1.67–11.66). Fathers whose workplaces were far from home and who were mostly absent at night had 56% lower odds of involvement (AOR = 0.44, 95% CI: 0.22–0.90). Fathers with good knowledge had 2.84 times higher odds of involvement (AOR = 2.84, 95% CI: 1.78–4.51). Fathers with a positive attitude had 2.10 times higher odds of involvement (AOR = 2.10, 95% CI: 1.34–3.28). Fathers with good cultural beliefs had 2.22 times higher odds of involvement in child feeding (AOR = 2.22, 95% CI: 1.40–3.51) (Table 5). The Hosmer-Lemeshow goodness-of-fit test indicated that the model was adequately fitted to the data (*p* = 0.453). Multicollinearity was not a concern, as all variance inflation factor values for the included variables were below 10.

**Table 5:** Bivariable and multivariable binary logistic regression analysis for fathers’ involvement in child feeding among fathers with children aged 6–24 months in Chena district, Southwest Ethiopia.

Variables	Categories	Fathers’ involvement		COR (95% CI)	AOR (95% CI)	P-value
		Good	Poor			
Age of fathers (years)	18–30	106	54	5.77 (3.00–11.10)	1.48 (0.63–4.46)	0.35
	31–40	82	163	1.48 (0.79–2.76)	1.04 (0.47–2.28)	0.92
	41–50	55	99	1.63 (0.85–3.15)	1.10 (0.48–2.45)	0.83
	> 50	16	47	1	1	
Residence	Urban	54	24	3.72 (2.23–6.20)	1.07 (0.52–2.20)	0.86
	Rural	205	339	1	1	
Marital status	Monogamy	250	326	3.15 (1.20–4.83)	1.64 (0.66–4.08)	0.28
	Polygamy	9	37	1	1	
Educational level	No formal education	30	97	1	1	
	Primary education	55	178	1.00 (0.63–1.74)	0.78 (0.48–1.40)	0.42
	Secondary education	72	72	3.23 (2.23–6.40)	1.29 (0.67–2.51)	0.46
	College and above	102	16	20.6 (9.26–33.47)	4.41 (1.67–11.66)	0.003*
Occupation	Government employee	114	52	3.79 (1.68–8.53)	0.59 (0.19–1.78)	0.35
	Merchant	43	72	1.03 (0.45–2.37)	0.82 (0.28–2.34)	0.71
	Farmer	91	220	0.71 (0.33–1.56)	0.66 (0.25–1.79)	0.41
	Daily laborer	11	19	1	1	
Paternity	Biological	250	310	1	1	0.10
	Non-biological	9	53	0.21 (0.04–0.27)	0.50 (0.22–1.15)	
Source of information	Mass media	79	111	0.57 (0.32–1.05)	0.97 (0.23–2.20)	0.95
	Social media	6	17	0.29 (0.10–0.83)	0.49 (0.12–2.03)	0.31
	Health facility	92	130	0.57 (0.32–1.03)	1.01 (0.45–2.25)	0.97
	Neighbor/community	51	80	0.51 (0.27–0.97)	1.21 (0.51–2.84)	0.66
	No information	31	25	1	1	
Monthly income (ETB)	< 1500	75	160	1	1	
	1501–2500	39	82	1.02 (0.63–1.62)	0.97 (0.56–1.71)	0.91
	2501–3500	45	72	1.33 (0.84–2.12)	1.01 (0.56–1.84)	0.98
	> 3500	100	49	4.35 (2.81–6.75)	1.37 (0.67–2.80)	0.39
Sex of youngest child	Male	160	167	1.90 (1.37–2.62)	0.98 (0.63–1.51)	0.93
	Female	99	196	1	1	
Workplace	Near residence, home at night	226	240	1	1	
	Far, sometimes away	18	53	0.36 (0.21–0.63)	0.76 (0.37–1.56)	0.46
	Far, mostly away	15	70	0.23 (0.13–0.41)	0.44 (0.22–0.90)	0.024*
Knowledge	Good	218	155	7.14 (4.81–10.57)	2.84 (1.78–4.51)	< 0.001*
	Poor	41	208	1	1	
Attitude	Positive	169	88	5.87 (4.13–8.33)	2.10 (1.34–3.28)	0.001*
	Negative	90	275	1	1	
Cultural belief	Good	201	132	6.07 (4.22–8.71)	2.22 (1.40–3.51)	< 0.001*
	Bad	58	231	1	1	

Note: *Significantly associated variables at *P* < 0.05.

## Discussion

This study assessed the level of fathers’ involvement in child feeding and its associated factors among fathers of children aged 6–24 months in Chena town, southwest Ethiopia. This study found that 41.6% of fathers were involved in child feeding. This low level of participation may reflect entrenched socio-cultural norms in Ethiopia, where childcare is traditionally viewed as the mother’s responsibility, and fathers are often preoccupied with income-generating activities outside the home. Such patterns underscore the persistent gendered division of labor, which can directly affect optimal infant and young child feeding practices and, consequently, child nutrition outcomes.

The proportion of fathers involved in child feeding is comparable to findings from Antsokiya Gemza District, North Shoa, Ethiopia (43.1%)^1^, and the National Capital Region of India, where 43% of first-time fathers were actively involved in infant and young child feeding^30^. These similarities may reflect comparable geographic contexts and the implementation of community-based nutritional interventions, including counseling and outreach programs. However, the proportion in this study was lower than reports from Damot Woyde District, South Ethiopia (50.9%)^31^; rural southwestern Uganda (65.5%)^32^; and Abim District, Uganda (68%)^33^. The discrepancies may be explained by differences in community nutrition programs, socio-cultural norms, geographic context, and the predominant role of mothers in childcare, while fathers spend significant time away for income-generating activities.

Fathers with a college education or above had 4.41 times higher odds of being involved in child feeding than those with no formal education (AOR = 4.41, 95% CI: 1.67–11.66), consistent with studies in Antsokiya Gemza district^1^, Southern Ethiopia, and Amuru District, Northern Uganda^34,35^. Education may empower fathers with critical knowledge, decision-making capacity, and openness to non-traditional roles in childcare. This finding suggests that improving male education and literacy could indirectly promote better child nutrition by enhancing paternal engagement.

Workplace location also influenced involvement. Fathers working far from home and mostly absent at night were 56% less likely to participate in child feeding (AOR = 0.44, 95% CI: 0.22–0.90). This is consistent with evidence from Juba, South Sudan^36^, and Amuru District, Uganda^30^, where work-related absences reduced male support in infant feeding. Similarly, studies in Uganda reported that men working away from their households were less likely to engage in child feeding activities^35^. This emphasizes that structural factors such as work schedules, commuting, and employment type materially influence caregiving behaviors. Interventions aimed at increasing paternal involvement should therefore consider workplace flexibility and community support systems to mitigate time constraints.

Good knowledge was strongly associated with involvement (AOR = 2.84, 95% CI: 1.78–4.51), corroborating studies from Antsokiya Gemza District and Amuru District^1,31^. Awareness generated through health extension programs and community nutrition dialogues likely enhances fathers’ understanding and encourages supportive practices. Comparable results were reported in the National Capital Region of India and Western Kenya, where fathers receiving health and nutrition information demonstrated higher involvement^30,34^.

Positive attitudes also significantly increased fathers’ involvement (AOR = 2.10, 95% CI: 1.34–3.28), in agreement with studies from Antsokiya Gemza District and Northern Ghana^1,35^. Behavioral change communication strategies, including the Infant and Young Child Feeding Quick Reference Guide in Ethiopia^37^and initiatives such as Alive & Thrive^4^, have demonstrated that fostering positive emotions and attitudes promotes paternal engagement. Similar associations were observed in Southern England, where fathers with positive attitudes were more active in child feeding^38^. This demonstrates that informational interventions alone are insufficient unless accompanied by attitude-shaping strategies, including behavior change communication, counseling, and culturally relevant education. These findings resonate with broader evidence indicating that awareness, motivation, and perceived self-efficacy are interdependent factors driving caregiving behavior, suggesting the need for multifaceted interventions targeting both cognitive and affective domains.

Finally, supportive cultural beliefs were associated with higher involvement (AOR = 2.22, 95% CI: 1.40–3.51). This finding aligns with studies from Antsokiya Gemza District^1^and Karongi District, Rwanda, where couple communication, shared decision-making, and gradual shifts in gender norms facilitated male engagement in child feeding^39^. Conversely, in Amuru District, Northern Uganda, cultural beliefs were reported as barriers to fathers’ participation^40^. These results highlight the dual role of culture as both a barrier and facilitator, indicating that interventions must be context-sensitive, leveraging positive norms while challenging restrictive practices.

Collectively, these findings reveal that fathers’ involvement in child feeding is determined by an interplay of individual (knowledge, attitude, education), structural (workplace, time), and socio-cultural factors. Interventions to enhance paternal engagement should therefore be holistic, combining education, community-based programming, workplace policies, and culturally tailored messaging. Engaging fathers alongside mothers can improve complementary feeding practices, dietary diversity, and ultimately child health outcomes.

This study has some limitations that should be considered when interpreting the findings. First, the cross-sectional design precludes establishing causal relationships between fathers’ involvement in child feeding and the associated factors identified. Second, the study relied on self-reported data, which may be subject to recall bias and social desirability bias, potentially leading to overreporting of socially acceptable behaviors despite efforts to minimize this during data collection. Third, although the measurement items were informed by existing literature, the absence of a universally standardized and fully validated tool for assessing fathers’ involvement in child feeding may have affected measurement reliability and limits generalizability. Cultural context and local norms may also influence how involvement is perceived and reported. Finally, although monthly household income was collected as a proxy for economic status, household food insecurity, a recognized determinant of fathers’ involvement, was not directly assessed, limiting the ability to examine its influence. Additionally, cultural beliefs are complex and may be better explored using mixed-methods approaches; in this study, they were measured using structured questionnaires, which may not fully capture nuanced perspectives. Future research should incorporate formal measures of food security and consider qualitative or mixed-methods designs to provide a more comprehensive understanding of factors influencing fathers’ participation in child feeding.

Despite these limitations, this study contributes important evidence from a setting where fathers’ roles in child feeding remain underexplored. By identifying modifiable individual, structural, and socio-cultural factors, the findings provide actionable guidance for nutrition programs, health extension services, and policymakers seeking to strengthen paternal engagement and improve child nutrition and survival outcomes.

## Conclusion

Fathers’ involvement in child feeding in Chena District was low, with only 41.6% demonstrating good engagement. Higher educational attainment, work location close to home, good knowledge, positive attitudes, and supportive cultural beliefs were associated with better involvement. These findings highlight the importance of promoting fathers’ participation through targeted education, behavior change communication, and community-based nutrition interventions. Integrating male partners alongside mothers in nutrition programs, prioritizing house-to-house outreach for fathers who spend limited time at home, and providing counseling on effective communication and participation even when away may help enhance engagement. Institutionalizing couple-focused counseling on infant and young child feeding at health facilities and strengthening support from the District Health Office through community conversations, health education, and behavior change communication could further encourage male participation. Strengthening fathers’ involvement may be associated with improved complementary feeding practices and child nutrition outcomes, contributing to better child health and survival.

## Supplementary Information

Below is the link to the electronic supplementary material.

Supplementary Material 1

Supplementary Material 2

Supplementary Material 3

Supplementary Material 4

## Data Availability

the datasets used and/or analyzed during the current study are available from the corresponding author upon reasonable request.

## Abbreviations

AOR: Adjusted odds ratio
CF: Child feeding
CI: Confidence interval
HSTP: Health sector transformation plan
IYCF: Infant and young child feeding
SPSS: Statistical package for social science
