# Supplementary material for: Fathers’ involvement in child feeding and associated factors among fathers of children aged 6–24 months in Chena District, Southwest Ethiopia: a community-based cross-sectional study
Source: Sci Rep. 2026 Feb 15;16:9142. doi: 10.1038/s41598-026-40365-1 (PMC12996599; doi:10.1038/s41598-026-40365-1)
Supplement: Supplementary file 1 — Supplementary Material 1 [file 41598_2026_40365_MOESM1_ESM.docx]

Knowledge of fathers about involvement in child feeding among fathers with children aged 6–24 months in Chena district, Southwest Ethiopia.

| **Knowledge Variable** | **Category** | **Frequency (n)** | **Percent (%)** |
| --- | --- | --- | --- |
| Age to start complementary feeding | Before six months | 108 | 17.4 |
|  | At six months | 330 | 53.1 |
|  | After six months | 94 | 15.1 |
|  | I don't know | 90 | 14.5 |
| Duration of breastfeeding | Up to one year | 122 | 19.6 |
|  | Up to two years or beyond | 324 | 52.1 |
|  | Up to six months only | 85 | 13.7 |
|  | I don’t know | 26 | 4.2 |
| Proper nourishment to lactating mother | Only to keep health | 200 | 32.2 |
|  | Health + enough breast milk | 361 | 58.0 |
|  | I don’t know | 61 | 9.8 |
| Importance of complementary food at recommended age | Reduce breastfeeding frequency | 122 | 19.6 |
|  | Breast milk alone not enough | 474 | 76.2 |
|  | I don’t know | 26 | 4.2 |
| Food items to include in child diet | Only grain, legume, nuts | 162 | 26.0 |
|  | Grain, legumes, nuts, vitamins, minerals, meat, eggs | 416 | 66.9 |
|  | Don’t know | 44 | 7.1 |
| Meal frequency with age | Decrease | 200 | 32.2 |
|  | Increase | 361 | 58.0 |
| Fathers’ awareness on feeding responsibility | Don’t know | 61 | 9.8 |
|  | Mother’s responsibility | 96 | 15.4 |
|  | Preparing meal | 155 | 24.9 |
|  | Financial support | 159 | 25.6 |
|  | Gardening | 80 | 12.9 |
|  | Preparing meal + financial support | 19 | 3.1 |
|  | Preparing meal + gardening | 36 | 5.8 |
|  | Financial + gardening | 29 | 4.7 |
|  | Preparing meal + financial + gardening | 12 | 1.9 |
|  | Don’t know | 36 | 5.8 |
| Facilitates/smoothen feeding environment | Not sharing mother’s workload | 89 | 14.3 |
|  | Understanding child response | 222 | 35.7 |
|  | Providing social & emotional support | 127 | 20.4 |
|  | Understanding child response + social/emotional support | 152 | 24.4 |
|  | Don’t know | 32 | 5.1 |
| Consequences of poor nutrition | Normal weight | 57 | 9.2 |
|  | Stunting | 194 | 31.2 |
|  | Wasting | 107 | 17.2 |
|  | Under/overweight | 97 | 15.6 |
|  | Stunting + wasting | 36 | 5.8 |
|  | Stunting + under/overweight | 39 | 6.3 |
|  | Wasting + under/overweight | 48 | 7.7 |
|  | Stunting, wasting, under/overweight | 17 | 2.7 |
|  | Don’t know | 27 | 4.3 |
| Overall knowledge | Good knowledge | 373 | 60.0 |
|  | Poor knowledge | 249 | 40.0 |
