# Supplementary material for: Fathers’ involvement in child feeding and associated factors among fathers of children aged 6–24 months in Chena District, Southwest Ethiopia: a community-based cross-sectional study
Source: Sci Rep. 2026 Feb 15;16:9142. doi: 10.1038/s41598-026-40365-1 (PMC12996599; doi:10.1038/s41598-026-40365-1)
Supplement: Supplementary file 2 — Supplementary Material 2 [file 41598_2026_40365_MOESM2_ESM.docx]

Fathers’ involvement in child feeding among fathers of children aged 6–24 months in Chena District, Southwest Ethiopia.

| **Variables** | **Yes, always n (%)** | **Yes, often n (%)** | **Yes, sometimes n (%)** | **Never n (%)** |
| --- | --- | --- | --- | --- |
| Father discusses and decides with mother to continue breastfeeding up to two years and beyond | 91 (14.6) | 237 (38.1) | 278 (44.7) | 16 (2.6) |
| Father discusses and decides to start complementary feeding at six months | 132 (21.2) | 207 (33.3) | 273 (43.9) | 10 (1.6) |
| Father facilitates nutritional care to lactating mothers | 101 (16.2) | 200 (32.2) | 305 (49.0) | 16 (2.6) |
| Father encourages mother to breastfeed child alternately on each breast | 51 (8.2) | 83 (13.3) | 326 (52.4) | 162 (26.0) |
| Father farms multiple nutritious food items in garden | 84 (13.5) | 166 (26.7) | 294 (47.3) | 78 (12.5) |
| Father motivates child by gesturing while breastfeeding | 89 (14.3) | 142 (22.8) | 273 (43.9) | 118 (19.0) |
| Father buys nutritious and diversified food items from shop or market | 76 (12.3) | 163 (26.2) | 257 (41.3) | 126 (20.3) |
| Father feeds child in response to child’s cues | 83 (13.3) | 127 (20.4) | 272 (43.7) | 140 (22.5) |
| Father assists mother with household chores | 74 (11.9) | 134 (21.5) | 270 (43.4) | 144 (23.2) |
| Father increases minimum acceptable diet as child grows | 79 (12.7) | 135 (21.7) | 299 (48.1) | 109 (17.5) |
| Father prepares solid, semi-solid, or soft food for child | 56 (9.0) | 131 (21.1) | 332 (53.4) | 103 (16.6) |
| Father feeds bottled breast milk when mother is away | 25 (4.0) | 64 (10.3) | 290 (46.6) | 243 (39.1) |
| Father assigns elder child to gesture while breastfeeding | 4 (0.6) | 37 (5.9) | 536 (86.2) | 45 (7.2) |

| **Involvement of fathers** | **Frequency** | **Percentage** |
| --- | --- | --- |
| Good involvement | 259 | 41.6 |
| Poor involvement | 363 | 58.4 |
