# Supplementary material for: Fathers’ involvement in child feeding and associated factors among fathers of children aged 6–24 months in Chena District, Southwest Ethiopia: a community-based cross-sectional study
Source: Sci Rep. 2026 Feb 15;16:9142. doi: 10.1038/s41598-026-40365-1 (PMC12996599; doi:10.1038/s41598-026-40365-1)
Supplement: Supplementary file 3 — Supplementary Material 3 [file 41598_2026_40365_MOESM3_ESM.docx]

Attitude of fathers toward involvement in child feeding among fathers with children aged 6–24 months in Chena district, Southwest Ethiopia.

| **Statements** | **Strongly agree** | **Agree** | **Neutral** | **Disagree** | **Strongly disagree** |
| --- | --- | --- | --- | --- | --- |
|  | Frequency (%) | Frequency (%) | Frequency (%) | Frequency (%) | Frequency (%) |
| Father feels satisfaction when mother continues breastfeeding up to 2 years or beyond | 78 (12.5%) | 102 (16.4%) | 233 (37.5%) | 175 (28.1%) | 34 (5.5%) |
| Lactating mother cannot manage household chores without support | 75 (12.1%) | 213 (34.2%) | 193 (31.0%) | 123 (19.8%) | 18 (2.9%) |
| Father’s role is important as mothers in child feeding | 103 (16.6%) | 142 (22.8%) | 225 (36.2%) | 136 (21.9%) | 16 (2.6%) |
| Father politely discusses with mother about challenges and benefits of child feeding | 78 (12.5%) | 132 (21.2%) | 143 (23.0%) | 196 (31.5%) | 73 (11.7%) |
| Fathers should buy child’s nutritional items | 80 (12.9%) | 121 (19.5%) | 218 (35.0%) | 167 (26.8%) | 36 (5.8%) |
| Father feels happy when supporting wife in child feeding | 76 (12.2%) | 164 (26.4%) | 186 (29.9%) | 159 (25.6%) | 37 (5.9%) |
| Father asks wife how and when to prepare child’s food | 77 (12.4%) | 115 (18.5%) | 199 (32.0%) | 184 (29.6%) | 47 (7.6%) |
| Fathers can bathe children and wash clothes without shame | 51 (8.2%) | 132 (21.2%) | 187 (30.1%) | 179 (28.8%) | 73 (11.7%) |

| **Attitude** | **Frequency (n)** | **Percent (%)** |
| --- | --- | --- |
| Positive | 257 | 41.3 |
| Negative | 365 | 58.7 |
