# Supplementary material for: Fathers’ involvement in child feeding and associated factors among fathers of children aged 6–24 months in Chena District, Southwest Ethiopia: a community-based cross-sectional study
Source: Sci Rep. 2026 Feb 15;16:9142. doi: 10.1038/s41598-026-40365-1 (PMC12996599; doi:10.1038/s41598-026-40365-1)
Supplement: Supplementary file 4 — Supplementary Material 4 [file 41598_2026_40365_MOESM4_ESM.docx]

Cultural belief of fathers toward father’s involvement in child feeding among fathers with children aged 6–24 months in Chena district, Southwest Ethiopia.

| **Variables** | **Categories** | **Frequency (n)** | **Percentage (%)** |
| --- | --- | --- | --- |
| Community discourages father when he involves in child feeding | Yes | 212 | 34.1 |
|  | No | 410 | 65.9 |
| Partner/wife shows disrespect to father when he involves in child feeding | Yes | 202 | 45.3 |
|  | No | 340 | 54.7 |
| Neighbors demoralize father when he involves in child feeding | Yes | 295 | 47.4 |
|  | No | 327 | 52.6 |
| Father’s mother and relatives demoralize him when he involves in child feeding | Yes | 276 | 44.4 |
|  | No | 346 | 55.6 |
| Community believes that child feeding is mother’s role | Yes | 304 | 48.9 |
|  | No | 318 | 51.1 |
| **Overall Cultural Belief** | Good | 333 | 53.5 |
|  | Bad | 289 | 46.5 |
